# Impact of Co-Infections on COVID-19: Special Issue Editorial

**DOI:** 10.3390/v17101345

**Published:** 2025-10-07

**Authors:** Telugu Akula Narasaraju, Sunil More, Yee-Joo Tan

**Affiliations:** 1 Department of Microbiology, Adichunchanagiri Institute of Medical Sciences, School of Natural Sciences, Center for Research and Innovation, Adichunchanagiri University, BG Nagara, Mandya 571448, Karnataka, India; 2 Department of Veterinary Pathobiology, Oklahoma State University, Stillwater, OK 74074, USA; 3 Infectious Diseases Translational Research Programme, Department of Microbiology and Immunology, Yong Loo Lin School of Medicine, National University of Singapore, Singapore 117545, Singapore

Since its emergence in December 2019, the COVID-19 pandemic has culminated in over 7.0 million deaths worldwide. Numerous clinical reports have shown that secondary superinfections with a wide range of viral, bacterial, and fungal pathogens contributed to fatal outcomes in COVID-19. The interaction and interplay between SARS-CoV-2 and microbes involved in co-infection, host defense, and immune response contributed to the outcome of the COVID-19 disease. This Special Issue provides critical insights into how co-infections shape COVID-19 outcomes. It focuses on signaling events involved in pathogen–pathogen and pathogen–host interactions, as well as clinical reports, epidemiological studies, and work on animal models.

Given that bacterial co-infection is fairly common during respiratory virus infections and can significantly impact a patient’s outcome, this Special Issue has three interesting papers covering different aspects related to bacterial co-infection in COVID-19 patients. Said et al. [1] investigated the effect of bacterial co-infections during the SARS-CoV-2 pandemic and compared infection profiles and fatalities in three phases, including before, during, and after COVID-19 vaccination. These studies found increased fatal outcomes in bacterial co-infections compared to non-co-infected patients. In addition, after SARS-CoV-2 vaccination, the infections and fatalities were significantly reduced. Among different bacterial co-infections, these studies found that Methicillin-resistant *S. aureus* was most dominant, followed by *E. coli* co-infections. These findings highlight that vaccination provides co-protection from secondary bacterial infections. Next, Papic et al. [2]. focused on the effect of corticosteroid therapy and its associated risk in bacterial superinfections in COVID-19 patients. The authors performed a retrospective study by analyzing bacterial superinfections among COVID-19 patients who were treated with corticosteroids. The bacterial coinfections were assessed by evaluating bacteremia for circulating bacteria in the bloodstream. The authors compared the low and progressive increase in the corticosteroid doses treatment in severely ill COVID-19 patients on the bacteremia and fatal outcomes. This study highlights that high and very high corticosteroid doses were associated with an increased risk of life-threatening complications and comorbidity burden in COVID-19 patients, while administration of low doses of corticosteroids was a better choice of treatment. Finally, Casale et al. [3]. highlighted that superinfections with Carbapenem-resistant *Acinetobacter baumannii* and carbapenemase-producing Enterobacterales were most common among adult COVID-19 patients. Although the incidence of KPC-producing *Klebsiella pneumoniae* and/or carbapenem-resistant *Acinetobacter baumannii* was high during hospitalization, these co-infections were not associated with increased mortality.

Importantly, this Special Issue also has a series of papers covering the phenomenon of viral interference in SARS-CoV-2 co-infections. In Svyatchenko et al. [4] in vitro (Vero E6 cells) and in vivo (Syrian hamster) studies were performed to evaluate the disease outcome in SARS-CoV-2 and enterovirus vaccine strain LEV8 (LV8) or enterovirus A71 (EV-A71) co-infections. These co-infection studies demonstrated that LV8 or EV-A71 co-infections suppressed SARS-CoV-2 replication in vitro and significantly enhanced SARS-CoV-2 viral clearance. Further, co-infection with LV8 or EV-A71 was shown to reduce clinical manifestations and decrease pathologic lesions in the lungs when compared to SARS-CoV-2 alone-infected animals. The authors reported that protection during co-infection with enteroviruses was due to an increased antiviral immune response against SARS-CoV-2-mediated lung pathology. Teluguakula et al. [5]. described the association of influenza co-infections in SARS-CoV-2-infected patients and discussed how this co-infection affected the survival and replication of both viruses. This review article highlighted two possible scenarios, where both viruses could compete with each other for survival and thus could trigger virus interference used to suppress the growth of the other virus. In a second scenario, both viruses could exhibit a synergistic effect and exacerbate lung pathology, thus worsening the infection.

While the above papers highlight the complex interplay between different viruses, known to cause acute infections, the next paper by Caciagli et al. [6]. highlighted the association of cytomegalovirus (CMV) reactivation in COVID-19-associated invasive pulmonary aspergillosis (CAPA). The aspergillus superinfections led to poor outcomes in critically ill COVID-19 patients. The authors found that 40% of CAPA patients were positive for blood CMV levels. However, CAPA + CMV co-infections did not yield high mortality compared to CAPA patients alone, as evaluated by a 90-day mortality study. The authors further identified that CMV infection was independent of prior immunosuppression in CAPA patients, and CMV infection appeared to reduce only the lengths of ICU stay for CAPA patients but was not associated with mortality.

Finally, the paper by Erickson et al. [7] focused on the evaluation of pentasilver hexaoxoiodate (Ag_5_IO_6_) as a broad antiviral drug. This group tested the antiviral effect of pentasilver hexaoxoiodate (Ag_5_IO_6_) against SARS-CoV-2 in vitro using cell lines including H1HeLa, RAW 264.7, LLC-MK2, Vero E6, and Vero E6. In vitro treatment with Ag_5_IO_6_ inhibited SARS-CoV-2 viruses’ (original and omicron) replication and also murine norovirus completely. However, Ag_5_IO_6_ showed partial inhibition of adenovirus, but it completely failed to inhibit the poliovirus. These important studies identified Ag_5_IO_6_ as a potent antiviral agent. Further study may reveal whether Ag_5_IO_6_ can inhibit the replication of multiple viruses in vivo and determine whether it is useful for treating patients with co-infection of two or more viruses.

This Special Issue provides critical insights into how co-infections influence disease outcomes in COVID-19. The articles published are based on clinical studies, retrospective analysis, and animal models, which provide valuable insights into understanding how various microbial pathogens contribute to lung pathophysiology in COVID-19. The co-existence of multiple pathogens in the same lung microenvironment could lead to competition for survival or could function synergistically, worsening the disease outcome. This Special Issue thus introduces critical aspects that help scientists to better understand the pathogenesis in COVID-19-associated co-infections.
